# The role of cancer-associated fibroblasts in the invasion and metastasis of colorectal cancer

**DOI:** 10.3389/fcell.2024.1375543

**Published:** 2024-07-30

**Authors:** Jinjin Yin, Wenting Zhu, Senling Feng, Pengke Yan, Shumin Qin

**Affiliations:** ^1^ Department of Pharmacy, Guangdong Provincial Key Laboratory of Major Obstetric Diseases, Guangdong Provincial Clinical Research Center for Obstetrics and Gynecology, The Third Affiliated Hospital, Guangzhou Medical University, Guangzhou, China; ^2^ International Institute for Translational Chinese Medicine, School of Pharmaceutical Science, Guangzhou University of Chinese Medicine, Guangzhou, China; ^3^ Department of Gastroenterology, The Second Affiliated Hospital of Guangzhou University of Chinese Medicine, Guangzhou, China; ^4^ Guangdong Provincial Key Laboratory of Clinical Research on Traditional Chinese Medicine Syndrome, Guangzhou, China

**Keywords:** cancer-associated fibroblasts, colorectal cancer, heterogeneity, invasion, metastasis

## Abstract

Colorectal cancer (CRC) is the third most common cancer and has ranked the third leading cause in cancerassociated death globally. Metastasis is the leading cause of death in colorectal cancer patients. The role of tumor microenvironment (TME) in colorectal cancer metastasis has received increasing attention. As the most abundant cell type in the TME of solid tumors, cancer-associated fibroblasts (CAFs) have been demonstrated to have multiple functions in advancing tumor growth and metastasis. They can remodel the extracellular matrix (ECM) architecture, promote epithelial-mesenchymal transition (EMT), and interact with cancer cells or other stromal cells by secreting growth factors, cytokines, chemokines, and exosomes, facilitating tumor cell invasion into TME and contributing to distant metastasis. This article aims to analyze the sources and heterogeneity of CAFs in CRC, as well as their role in invasion and metastasis, in order to provide new insights into the metastasis mechanism of CRC and its clinical applications.

## 1 Introduction

Colorectal cancer (CRC) is one of the most common malignant tumors of the digestive tract in the world. According to the global Cancer statistics in 2020, the new cases of CRC rank the third among malignant tumors (Sung et al., 2021). Metastasis is the primary cause of cancer-related mortality in CRC patients (Siegel et al., 2017). It is well known that tumor metastasis is a complex multi-stage, multi-step process that is closely influenced by the tumor microenvironment (TME). TME is a complex tissue environment composed of the extracellular matrix (ECM) and various types of stromal cells, such as cancer-associated fibroblasts (CAFs), macrophages, inflammatory cells and mesenchymal stem cells, and CAFs interacts with all other cells in the TME through direct cell contact and cytokine secretion, thereby stimulating tumor progression and ultimately metastasis. Therefore, CAF is considered a potential new target for cancer therapy (Sahai et al., 2020). CAFs are activated fibroblasts, a major component of TME, accounting for nearly 70% of cells in tumor tissue. These cells are highly heterogeneous in origin, coming from different tissues or cells, and currently lack specific biomarkers. Although more studies have been conducted, the source remains undetermined, and their functions differ greatly depending on their origin (Chen and Song, 2019). A large body of evidence favors the pro-tumorigenic ability of CAFs, which can directly promote tumor cell proliferation and invasion, indirectly affect the peripheral ECM, the vascular system, and modulate immune function to promote tumor progression (Biffi and Tuveson, 2021). However, it has also been recently suggested that CAFs also have tumor suppressive effects under certain circumstances (Wang et al., 2021). And research has shown that their existence is correlated with prognosis, and they are already under evaluation as a possible target for treatment (Chen et al., 2021b).

In view of the fact that metastasis is the main cause of CRC-related deaths and the key role of CAFs in metastasis, therefore, in this review, we focused on the origin and heterogeneity of CAFs in CRC, biomarkers, and elaborated their roles in CRC invasion and metastasis, with the aim of providing new ideas for the study of the metastatic mechanism and clinical application of CAFs in CRC.

## 2 Origin and heterogeneity of CAFs in CRC

### 2.1 Cellular origin of CAFs in CRC

**FIGURE 1:**
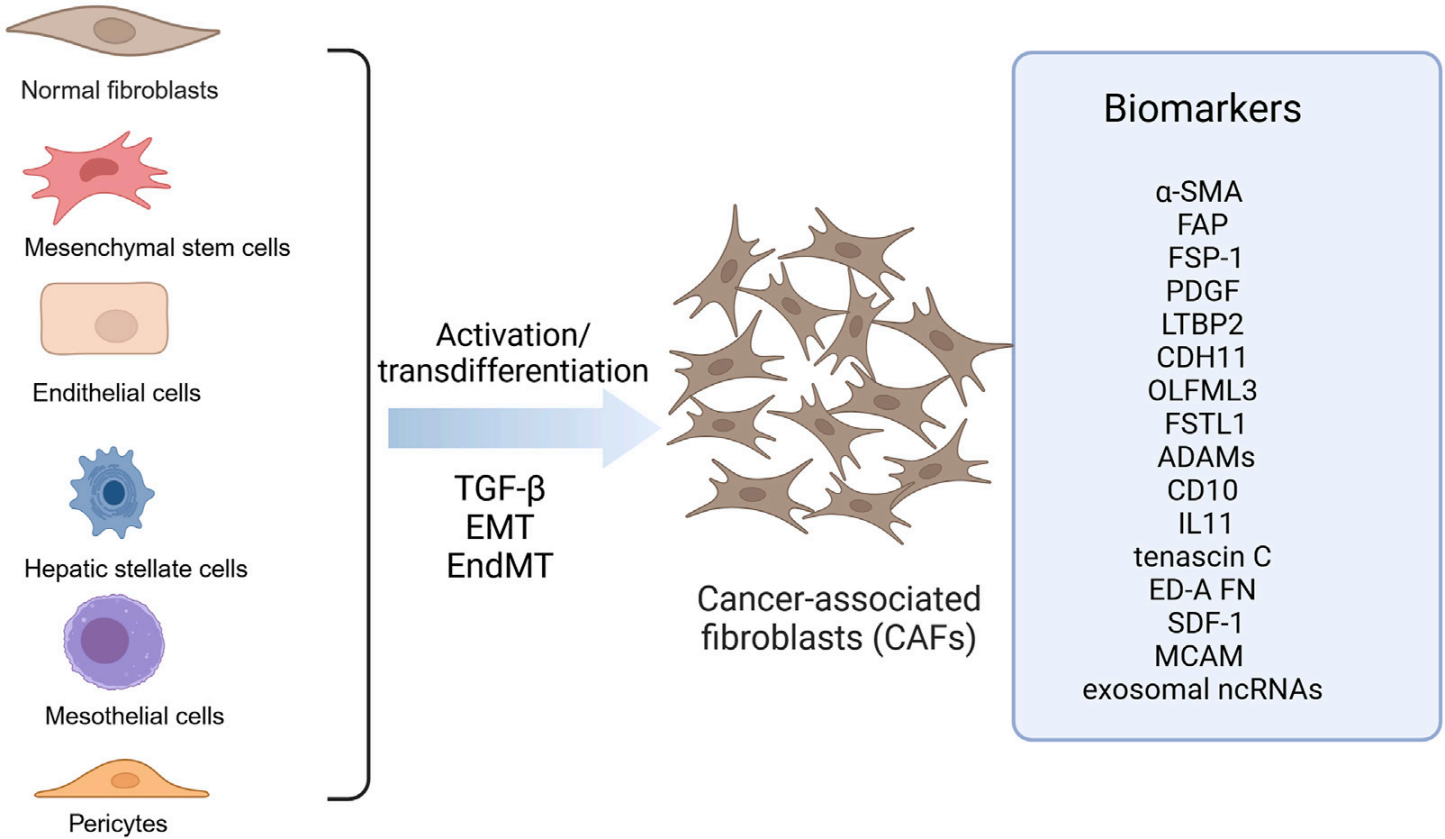
Origin of CAFs in colorectal cancer. CAFs in colorectal cancer are derived from diverse precursor cells through activation or transdifferentiation, including fibroblasts, mesenchymal stem cells, endothelial cells, hepatic stellate cells, mesothelial cells, pericytes. These CAFs express different biomarkers like a-SMA, FAP and FSP-1. ADAMS, A disintegrin and metalloproteinases; a-SMA, alpha-smooth muscle actin; CAFs, Cancer-associated fibroblasts; CDH11, Cadherin 11; ED-A FN, Fibronectin ED-A domain; FAP, Fibroblast activation protein α; FSP-1, Fibroblast specific protein-1; FSTL1, Follistatin-like 1; LTBP2, Latent TGF-b–binding protein 2; MCAM, Melanoma cell adhesion molecule; OLFML3, Olfactomedin-like 3; PDGF, Platelet-derived growth factor receptor; SDF-1, Stromal cell-derived factor 1. Image created with BioRender.com, with permission.

Fibroblasts are a major cell type in the stroma, present in almost all organs and normal tissues, and contribute to inflammation and fibrosis during wound healing. CAFs are activated fibroblasts, which are the main components of TME. CAFs are distinct from normal fibroblasts in terms of structure and function. They are larger with serrated nuclei and branched cytoplasm, and possess greater proliferative, migratory and secretory abilities as well as increased metabolic activity (Glabman et al., 2022). It has been shown that CAFs can be derived from 1) fibroblasts, 2) mesenchymal stem cells, 3) epithelial-mesenchymal transition (EMT), 4) endothelial-mesenchymal transition (EndMT) of resident endothelial cells, and 5) transformation of adipocytes, pericytes, and smooth muscle cells (Rimal et al., 2022). Its highly heterogeneous cellular origin determines the heterogeneity of the CAFs population, which affects their phenotype and function. In CRC, CAFs are mainly derived from peripheral fibroblasts and bone marrow-derived mesenchymal stem cells (Figure 1). However, the underlying molecular mechanisms governing the differentiation of precursor cells into CAFs remain poorly understood.

#### 2.1.1 Fibroblasts

In CRC, fibroblasts are the predominant source of CAFs. Researches have demonstrated that the integrin α-v-β6 expressed by tumor cells is capable of activating latent forms of transforming growth factor (TGF)-β, a well-known stimulator that induces the differentiation of quiescent fibroblasts into CAFs in TME (Hawinkels et al., 2014). Another TGF superfamily member, Nodal, has recently been shown to be positively correlated with alpha-smooth muscle actin (α-SMA) expression in CRC tissues, which can be secreted by tumor cells and promotes the transformation of normal fibroblasts into CAFs through the activation of the TGF-β/Smad/Snail pathway, thereby promoting CRC cell proliferation (Li et al., 2019). Some evidence also suggests that Snail-positive fibroblasts have CAF properties, further supporting that Snail is an important regulator of CAF formation from fibroblasts. And snail is a downstream target gene of TGF-β that mediates the tumor-promoting effects of TGF-β (Moon et al., 2017), and this gene is also a key factor in the tumor-promoting effects of fibroblasts in CRC (Herrera et al., 2014), so it is reasonable to hypothesize that Nodal promotes the progression of CRC by mediating the formation of CAFs through the Snail signaling pathway. In addition to Nodal, interleukin (IL) 34 overexpression in CRC cells is also involved in the transition of normal fibroblasts to CAFs (Franze et al., 2020). Except for CRC cells, the tumor mesenchyme also has a key role in the differentiation of fibroblasts into CAFs. For example, increased expression of matrix metalloproteinase inhibitor-1 (TIMP-1) promotes the formation of CAFs of fibroblast origin in CRC (Gong et al., 2013). Additionally, Dickkopf-3 (DKK-3), which is located in the stroma, has been demonstrated to activate the wingless/integrated (Wnt) and yes-associated protein/transcriptional co-activator with PDZ-binding motif (YAP/TAZ) signaling pathway, thereby leading to the formation of CAFs in CRC (Ferrari et al., 2019). Furthermore, deletion in protein kinase Cζ matrix has been found to be strongly correlated with CAFs stimulating tumor progression (Kasashima et al., 2021). Therefore, both tumor cells and mesenchyme in TME can promote the transformation of fibroblasts into CAFs.

#### 2.1.2 Mesenchymal stem cells (MSCs)

In addition to fibroblasts, MSCs derived from bone marrow have been extensively studied as another precursor of CAFs. These MSCs can migrate to the tumor stroma and differentiate into CAFs. It has been reported that CRC cells induce MSCs to differentiate into CAFs through cell-cell contact, which is mediated by Notch-Jagged1 and TGF-β/Smad signaling pathway (Peng et al., 2014). A recent study found that many α-SMA-positive CAFs in CRC arise from the proliferation of intestinal pericellular stromal cells (Lepr + cells), and Lepr is a well-established marker for perivascular mesenchymal cells, which support bone marrow hematopoietic stem cell maintenance (Kobayashi et al., 2022).

#### 2.1.3 Endothelial cells (ECs)

ECs can be converted into CAF by EndMT process. One study showed that invasive CRC cells induce the conversion of endothelial cells into CAFs through the upregulation and phosphorylation of tubulin-β3, which was mainly dependent on TGF-β stimulation (Wawro et al., 2018).

#### 2.1.4 Hepatic stellate cells

One study found that exosomes from CRC cells can stimulate hepatic stellate cells to become CAFs when liver metastasis of colon cancer occurs (C. Zhang et al., 2022). And a recent study also showed that CRC cells are able to interact with hepatic stellate cells to promote the secretion of stromal cell-derived factor 1 (SDF 1) and bind to C-X-C chemokine receptor type 4 (CXCR4) and induce the expression and secretion of TGF-β in CRC cells, which ultimately leads to the differentiation of hepatic stellate cells into CAFs, whereas the blockade of the CXCR4/TGF-β pathway inhibits the differentiation of hepatic CAFs and the metastasis of CRC to the liver (Tan et al., 2020).

#### 2.1.5 Mesothelial cells (MCs)

In CRC with deep infiltration near the serosa and peritoneal metastases, a considerable proportion of CAFs are derived from MCs. MCs gradually lose their mesothelial cell markers through mesothelial-to-mesenchymal transition (MMT) and approache myofibroblast morphology (Demuytere et al., 2020). Another histological observational study showed that MCs in locally advanced primary colorectal cancer also differentiated into CAFs via MMT (Type II EMT) (Gordillo et al., 2020), and RNA sequencing analysis showed that TGF-β was associated with MMT (Rynne-Vidal et al., 2017); therefore, whether MCs also differentiate by activating TGF-β needs to be further investigated.

#### 2.1.6 Pericytes

A study using single-cell RNA sequencing to analyze the transcriptome of primary tumors, matched liver metastases, and individual cells of peripheral blood from six patients with liver metastases from CRC revealed that contractile CAFs highly expressing pericyte-associated markers (e.g., RGS5 and CSPG4), largely were originate from pericytes (Che et al., 2021).

### 2.2 Biomarkers of CAFs in CRC

To identify and isolate CAFs from the fibroblast population throughout the body, markers such as α-SMA, fibroblast activation protein α (FAP), fibroblast specific protein-1 (FSP-1 or S100A4), vimentin, and platelet-derived growth factor receptor (PDGF) are commonly employed (Han et al., 2020; Nurmik et al., 2020). Despite the fact that CAFs originate from a variety of sources, they are not a unified group, but rather a subset of cells with varying spatial, phenotypic, and functional characteristics, thus, these biomarkers lack specificity. It has been proposed that a number of molecules may be used as biomarkers of CAFs in CRC. Proteomic analysis of normal fibroblasts and CAFs in colon tissue has revealed that latent TGF-b–binding protein 2 (LTBP2), cadherin 11 (CDH11), Olfactomedin-like 3 (OLFML3) and Follistatin-like 1 (FSTL1) may be used as biomarkers (Torres et al., 2013). Furthermore, increased expression of a disintegrin and metalloproteinases (ADAMs), such as ADAM9, ADAM10, ADAM12, and ADAM17, has been observed in CAFs found in colon tissues of CRC patients (Mochizuki et al., 2020). Additionally, CD10 (Zhu et al., 2016) and IL11 (Nishina et al., 2021) have also been suggested as potential markers of CAFs in CRC. In addition, a study of high-throughput differential secretome analysis of CAFs in colon cancer and non-cancer-activated bone marrow-derived MSC revealed that tenascin C, fibronectin ED-A domain and SDF-1 may also be biomarkers for CAFs in CRC (De Boeck et al., 2013). αSMA + CAFs express melanoma cell adhesion molecule (MCAM) derived from Lepr + cells, suggesting that MCAM can be used as a biomarker for αSMA + CAFs(Kobayashi et al., 2022). Interestingly, by performing the next-generation sequencing, a significant number of non-coding RNAs (ncRNAs) in exosomes were also found as potential biomarkers present in CAFs-derived exosomes (Herrera et al., 2018). It is worth noting that more studies are needed to validate whether these candidate molecules can be specific biomarkers for CAFs in CRC (Figure 1).

### 2.3 CAFs subtypes in CRC

The above biomarkers, such as α-SMA, FAP are commonly used to identify CAFs. However, labeling of CAFs is likely to be more complex, because it is now clear that CAFs are also characterized by increased heterogeneity (Koliaraki et al., 2017). CAFs may have multiple subtypes, which have different functions (Su et al., 2018). Recent progress in single-cell RNA-sequencing technologies has enabled detailed characterization of the complexity and heterogeneity of CAF subpopulations in multiple tumor types. Li et al. performed single-cell RNA sequencing of tumors and their microenvironments from samples derived from normal intestinal mucosa and CRC, and identified two distinct subtypes of CAFs, CAF-A and CAF-B. CAF-A cells express genes related to extracellular matrix remodeling and may be a separate subtype or an intermediate state between normal fibroblasts and CAF-B cells, while CAF-B cells express cytoskeletal genes and other known markers of fibroblasts (Li et al., 2017). In addition, Giguelay et al. identified two main subtypes of CAFs in patients with liver metastases from CRC, contractile CAFs (ctr-CAFs) and ECM remodeling/promoter angiogenesis CAFs (ECM-CAFs). And further analysis revealed that ECM remodeling/promoter angiogenesis CAFs were derived from intrinsic fibroblasts of the hepatic portal area (Giguelay et al., 2022). Chen LH et al. classified CAFs into three major subsets, including secretory CAFs, ECM-remodeling CAFs and contractile CAFs. Secretory CAFs highly express secretory proteins, such as various growth factors (e.g., Insulin-like growth factor 1(IGF1), Platelet-derived growth factor D (PDGFD), fibroblast growth factor (FGF) seven and Vascular endothelial growth factor B (VEGFB)) that mediate angiogenesis and cancer cell proliferation. The ECM-remodeling CAFs highly express ECM proteins (such as ECM collagens and fibronectin) and ECM proteases which alter ECM structure and assist tumor angiogenesis and metastasis. The contractile CAFs are enriched for genes involved in the regulation of cell contraction, suggesting some distinct phenotypes (Che et al., 2021). Chen SJ et al. identified five subtypes of CAFs from CRC animal model, then found that bifidobacterium adolescentis orchestrates CD143+ cancer-associated fibroblasts to suppress colorectal tumorigenesis (Chen et al., 2023). These studies revealed the diversity of CAFs in CRC and their complex functional properties, and that different subtypes of CAFs may originate from different precursor cells and perform different functions in TME, even have opposing capabilities such as tumor restraining vs tumor promoting (Table 1). Because of this, it is crucial to characterize CAFs as different subtypes based on their functional heterogeneity. In addition, while some studies have proposed potential precursor cells for specific CAFs subtype, their precise cellular identity remains elusive. Therefore, more studies need to continue to explore the origin, subtypes, and functions of CAFs in CRC and the relationship between them through the integration of cutting-edge techniques, including single-cell sequencing, spatial transcriptomics, lineage tracing method and advanced imaging techniques.

**TABLE 1:** CAFs subtypes in colorectal cancer.

Models/Methods	CAF subtypes	Biomarkers	Signatures/functions	Additional notes	References
● Patient samples ● scRNA-seq with Fluidigm C1 Platform	CAF-A	MMP2 DCN COL1A2	ECM remodeling	Intermediate state?	Li et al. (2017)
	CAF-B	α-SMA TAGLN PDGFA	Myofibroblast-like	Activated myofibroblasts	
● Patient samples ● scRNA-seq by 10× genomics	● ctr-CAFs	RGS5 MCAM MYH11	Contractile signature	Expressed blood vessel wall markers	Giguelay et al. (2022)
	● ECM-CAFs	MMP2 FAP PDGFRA	ECM remodeling	Featured genes involved in ECM remodeling and collagen production	
● Patient samples ● scRNA-seq by 10× genomics	secretory CAFs	● Growth factors: IGF1, PDGFD, FGF7,VEGFB ● Signal molecules: BMP4,WNT2B ● complements: C1S, C3 ● Chemokines: CCL2, CXCL12, CXCL14	● mediating angiogenesis and cancer cell proliferation ● maintaining cancer stem cell niche ● regulating tumor immunity and inflammation	Earlier developed subtype	Che et al. (2021)
	ECM-remodeling CAFs	● ECM collagens and fibronectin ● ECM proteases	● altering ECM structure and assisting tumor angiogenesis and metastasis	—	
	contractile CAFs	● Expressing genes involved in the regulation of cell contraction	—	Later developed subtype	
● Murine samples ● scRNA-seq by 10× genomics	CD143+ CAFs	FAP+ CD143+	● Exhibiting tumor suppressive effect	—	Chen et al. (2023)

This table summarizes the heterogeneity of CAFs, in CRC, detailing the CAF subtypes identified, their markers, and their putative functions. CD143, Cluster of differentiation 143; CXCL, C-X-C motif chemokine ligand; COL1A2, Collagen type Ⅰ alpha two chain; DCN, decorin; ECM, extracellular matrix; FAP, fibroblast activation protein; FGF7, Fibroblast growth factor 7; IGF, Insulin-like growth factor 1; MCAM, melanoma cell adhesion molecule; MMP, matrix metallopeptidase; MYH11, Myosin heavy chain 11; PDGF, Platelet-derived growth factor; PDGFR, platelet derived growth factor receptor; RGS5, Regulator-of-G-protein-signaling-5; TAGLN, transgelin; VEGFB, Vascular endothelial growth factor B.

## 3 The role of CAFs in CRC invasion and metastasis

**FIGURE 2:**
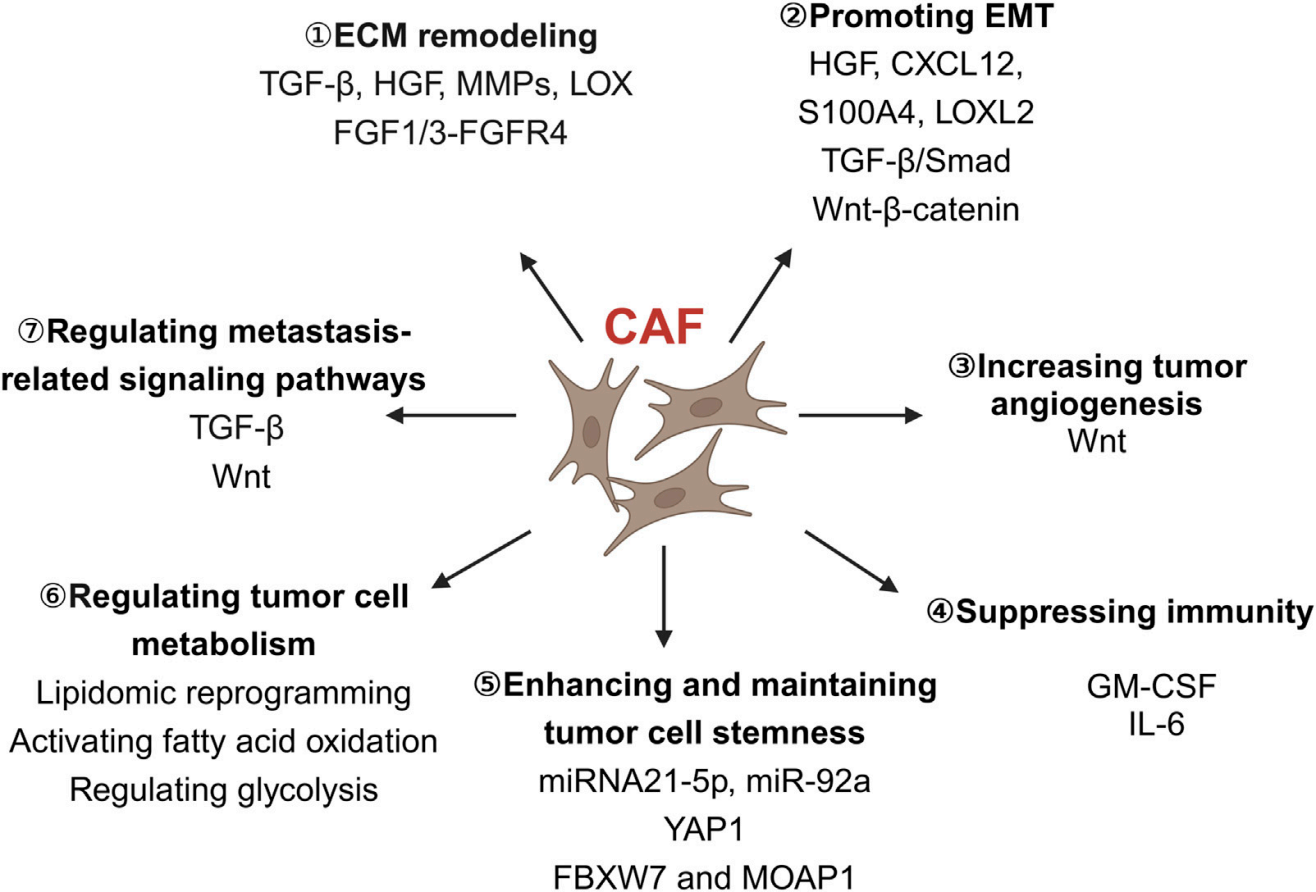
The role of CAFs in colorectal cancer invasion and metastasis. CAFs play a diverse role in colorectal cancer cell invasion and metastasis, including remodeling the ECM, promoting EMT, increasing tumor angiogenesis, suppressing immunity, enhancing and maintaining tumor cell stemness, regulating tumor cell metabolism and metastasis-related signaling pathways. CAFs, Cancer-associated fibroblasts; CXCL12, C-X-C chemokine ligand 12; ECM, Extracellular matrix; EMT, Epithelial-mesenchymal transition; FBXW7, F-Box and WD repeat domain containing 7; FGF, Fibroblast growth factor; GM-CSF, Granulocyte-macrophage colony-stimulating factor; HGF, Hepatocyte growth factor; IL-6, Interleukin-6; LOX, Lysyl oxidase; LOXL2, Lysyl oxidase-like 2; MMPs, Matrix metalloproteinases; MOAP1, Modulator of apoptosis 1; S100A4, S100 calcium-binding protein A4; TGF-β, Transforming growth factor-β; Wnt, Wingless/integrated; YAP1, Yes-associated protein 1. Image created with BioRender.com, with permission.

Comprehending the progression of events after cancer metastasis is of utmost importance, as it is often associated with a poor patient prognosis. The cancer metastasis cascade can be categorized into five steps: invasion, intravesation, circulation, extravasation, and colonization. Upon activation, CAFs can release a range of soluble factors that can enhance tumor invasion and metastasis (Linares et al., 2020), such as TGF-β, hepatocyte growth factor (HGF), epidermal growth factor (EGF), FGF and IL-6 cytokine (Owusu et al., 2017; Woolston et al., 2019; Zeng et al., 2017). These soluble factors can influence cancer invasiveness and metastasis by remodeling the ECM, regulating EMT in cancer cells, suppressing immunity, promoting angiogenesis, regulating cancer cell metabolism and metastasis-related signaling pathways (Figure 2).

### 3.1 CAFs remodel the ECM

TME is composed of cellular and non-cellular components, the latter of which is referred to as the ECM. ECM is made up of structural proteins, collagen, proteoglycans, hyaluronic acid, and glycosaminoglycans, which provide structural and signaling support for cells and tissues. Research has demonstrated that the structure and function of the ECM can be altered by cells in the TME, which has a significant impact on cancer metastasis (Li et al., 2020). The fundamental elements for ECM remodeling are TGF-β, HGF, and particular interleukins and Matrix metalloproteinases (MMPs). Studies on CRC have revealed that MMPs have a considerable impact on the ECM composition. MMPs are a family of enzymes that can disintegrate the basement membrane and almost all ECM elements. They are critical for the degradation of ECM involved in tumor cell invasion, metastasis, and angiogenesis. MMP-7 is the only and smallest enzyme in the MMPs family that is specifically expressed by tumor cells. Studies have indicated that it is more important than other members in the MMPs family in terms of breaking down and degrading the ECM and basement membrane on the tumor surface, as well as aiding angiogenesis, hastening tumor cell invasion and metastasis. And it is significantly expressed in liver metastasis or lymph node of colon cancer (Bi et al., 2013). A study showed that CAFs can upregulate MMP-7 expression in colon cancer cells through the FGF1/3-FGFR4 signaling pathway, resulting in the progression of CRC (Bai et al., 2015). That is, CAFs can create passages in the ECM by breaking down the matrix to enable CRC cells to migrate from the original site and metastasize. Several studies have shown that the stiffness of the ECM is positively correlated with cancer invasion and metastasis. In CRC, stiffer ECM increases activin A secretion in stromal cells, which in turn induces invasion via the EMT-related protein Snail (Bauer et al., 2020). CAFs can both degrade the ECM to create pathways for cancer cell migration, and increase ECM stiffness to further promote cancer invasion and metastasis (Bauer et al., 2020; Gkretsi and Stylianopoulos, 2018; Kalli and Stylianopoulos, 2018). This two seemingly contradictory process may be linked to the diverse origins and heterogeneity of CAFs(Asif et al., 2021). Furthermore, activated stromal cells have the ability to produce lysyl oxidase (LOX), which is responsible for the formation of collagen cross-linking. This in turn increases matrix stiffness and cancer cell invasion (Pankova et al., 2016; Sahai et al., 2020) and it has been found that lysyl oxidase-like 2 (LOXL2) is overexpressed in CAFs of CRC, which could be used as a marker for predicting the prognosis of colon cancer patients (Torres et al., 2015). Targeting the LOX oxidase produced by CAFs has been proposed as a potential approach to combat cancer metastasis (Liu et al., 2019). Overall, By understanding and modulating the complex interactions between CAFs, ECM, and CRC cells, researchers can develop novel approaches to disrupt the supportive environment that fosters CRC progression and metastasis.

### 3.2 CAFs promote EMT

Cancer metastasis involves a key step of ECM degradation, which is the acquisition of a mesenchymal phenotype. This is achieved through a process called EMT, where certain mesenchymal markers such as vimentin and fibronectin are upregulated, and epithelial junction proteins such as E-cadherin and occludin are downregulated. As a result, the intercellular adhesion junctions become weak, promoting the migration and invasion of tumor cells (Asif et al., 2021). Recent studies have indicated that CAFs can stimulate EMT of tumor cells which can facilitate distant metastasis. Normal human colon fibroblasts suppress EMT of colon cancer cells and Wnt-β-catenin signaling pathway activation, decreasing the migration and invasion capability of CRC cells. In contrast, CAFs are able to stimulate the secretion of Wnt4 and Wnt5 in CRC cells, thus activating the classical Wnt-β-catenin signaling pathway to induce EMT, which can result in the progression of CRC (Hu et al., 2019; Yang et al., 2020). Wanadi et al. demonstrated that CAFs can induce EMT of HT29 colon cancer cells through the secretion of HGF, thereby increasing their invasion and migration capabilities (Wanandi et al., 2021). In addition, Verification of other CAFs secreted factors involved in EMT has also been reported in CRC such as C-X-C chemokine ligand 12 (CXCL12), S100A4, and LOXL2 (Asif et al., 2021). Notably, CXCL12 (also known as SDF1) is a powerful activator of EMT which can induce this process via an over-activation of Wnt/β-catenin pathway via CXCR4/CXCL12 (Hu et al., 2014). And CXCR4/CXCL12 are believed to be potential therapeutic targets for CRC (Khare et al., 2021). S100A4 is a metastatic protein, also considered as an EMT-promoting protein, which is strongly associated with poor prognosis in CRC and promotes tumor cell progression and metastasis by down-regulating E-cadherin and modulating the EMT mesenchymal phenotype in epithelial cells (Fei et al., 2017). CAFs also stimulate EMT in colon cancer LOVO cells to promotes colon cancer metastasis through activating the focal adhesion kinase (FAK) signaling pathway by secreting LOXL2 (Xuefeng et al., 2020). Furthermore, CAFs can regulate EMT to promote metastasis by releasing exosomes, such as miRNA-181a, which can activate the TGF-β/Smad pathway and influence the EMT of colon cancer (Chao, 2020). Additionally, CAFs can directly transfer their own exosomes to CRC cells, which increases the level of miR-92a-3p in CRC cells. This can inhibit mitochondrial apoptosis and activate the Wnt/β-catenin pathway by suppressing F-Box and WD repeat domain containing 7 (FBXW7) and modulator of apoptosis 1 (MOAP1), thus promoting the EMT and metastasis of tumor cells (Hu et al., 2019). Similarly, exosomes miR-625-3p and LINC00659 also promote CRC cell migration, invasion, and EMT by inhibiting the Elav-like family member 2/WW domain-containing oxidoreductase (CELF2/WWOX) pathway and interacting directly with miR-342-3p, respectively (Zhang et al., 2022b; Zhou et al., 2021). In addition, IL-6 positively correlates with tumor TNM stage and is associated with depth of tumor infiltration and lymph node metastasis in CRC (Zeng et al., 2017). And it upregulates the expression of integrin β6 through the IL-6 receptor/signal transducer and activator of transcription (STAT)-3 signaling pathway, which promotes EMT and invasiveness of CRC cells (Sun et al., 2020). In summary, CAFs play a significant role in promoting CRC metastasis by inducing EMT through various molecular mechanisms. Understanding these processes could provide insights into potential therapeutic targets for CRC treatment.

### 3.3 CAFs increase tumor angiogenesis

Tumor cells are more proliferative than normal cells (De Palma et al., 2017). Therefore, tumor growth necessitates a heightened blood supply to meet the elevated demands for oxygen and nutrients. In human CRC, Wnt2 is preferentially upregulated in CAFs, which promotes metastasis and invasion. Wnt2 exhibits a pro-angiogenic role in the development of placental vasculature, stimulating angiogenesis within liver sinusoidal ECs and other ECs. In a comparative study between normal colon and CRC, CAFs were found to be the main producers of stromal Wnt2 in the stromal and epithelial compartments (Kramer et al., 2017). Wnt2 expression in CAFs resulted in the autocrine activation of canonical Wnt signaling and increased motility of fibroblasts, which positively affected the invasive and metastatic potential of CRC (Unterleuthner et al., 2020). Targeting Wnt2 or its signaling pathways could potentially be a promising strategy for CRC therapy.

### 3.4 CAFs suppress immunity

CAFs interact with most cells in the TME (Farhood et al., 2019), including various types of immune cells. Tumor-associated macrophages are the most abundant immune cells in the TME. Macrophages are usually classified into two groups: M1 and M2. The M1 phenotype is a pro-inflammatory cell type that has been shown to exhibit antitumor activity, while the M2 phenotype is an immunosuppressive cell type that promotes tumor progression. Schellerer et al. (2014) reported that CAFs express higher levels of intercellular adhesion molecule-1 (ICAM-1) and a higher affinity for attracting monocytes to infiltrate cancer tissues. Meiyun’research (Wang et al., 2020) further elucidates the role of CAFs in this process. They found that CAFs secrete exosomes rich in granulocyte-macrophage colony-stimulating factor (GM-CSF) and IL-6. These factors promote the differentiation of monocytes into M2 macrophages, and activate M2 macrophages to release chemokines and exosomes, thereby enhancing angiogenesis in CRC and promoting tumor metastasis. In conclusion, CAFs actively influence the TME by modulating the polarization of macrophages. This ultimately aids in the development and spread of CRC by stimulating angiogenesis and suppressing the immune system.

### 3.5 CAFs enhance and maintain tumor cell stemness

Cancer stem cells (CSCs) are closely related to tumor metastasis. CSCs are a class of tumor cells with the ability to self-renew and differentiate. Several studies have found that the tumor cell stemness is been increased by exosomes secreted from CAFs, which have been also found to be effectively taken up by tumor cells, thereby resulting in the promotion of proliferation, invasion, and metastasis of cancer cells (Chen et al., 2021a). For example, miRNA21-5p expressed in CAF-derived exosomes increases the proportion of CSCs and enhances the invasion and metastasis of cancer cells by regulating YAP1 protein. Similarly, exosomal miR-92a maintains the stemness of CRC cells and promotes their invasion and metastasis, and induces resistance to 5-FU/L-OHP by targeted inhibition of FBXW7 and MOAP1 (Hu et al., 2019). Therefore, It has been suggested that the treatment of cancer should not only focus on CSCs, but also inhibit the secretion of CAFs, in order to maximize the benefits for patients (Chen et al., 2021b).

### 3.6 CAFs regulate tumor cell metabolism

It has been recently discovered that CAFs can also regulate cancer cell metabolism (Li et al., 2021). Activated CAFs have been observed to promote metabolic switching in CRC cells, resulting in increased organ metastases as a result of glutamine depletion. Furthermore, CAFs undergo lipidomic reprogramming, resulting in increased production and secretion of fatty acids. These fatty acids are then taken up by CRC cells, enhancing their migratory capabilities (Gong et al., 2020). This process not only supports the growth of primary tumors but also contributes to the formation of distant metastases. CAFs also regulate the metabolism of CRC cells by activating fatty acid oxidation and regulating glycolysis (Peng et al., 2021). This shift in metabolism may give cancer cells the energy and resources they need to survive and proliferate, which can accelerate the disease’s progression. Excitingly, interventions targeting the metabolic activity of CAFs have shown promise in restraining CRC progression. For instance, the use of catechin to inhibit the aerobic glycolysis activity of CAFs has been found to inhibit the migration and invasion ability of colon cancer cell lines like HCT-116 and HT29 (Chen et al., 2022). These discoveries highlight the significant role of CAFs in shaping the metabolic landscape of CRC. Targeting the metabolic activities of CAFs presents promising avenues for therapeutic intervention in restraining CRC metastasis.

### 3.7 CAFs regulate metastasis-related signaling pathways

Some soluble factor secreted by CAFs play crucial roles in regulating cancer metastasis by modulating specific signaling pathways. One such factor is TGF-β, a key member of the TGF superfamily. TGF-β is known to play a pivotal role in mediating the interaction between tumor cells and CAFs. Research suggests that TGF-β can induce the differentiation of normal fibroblasts into CAFs, and these CAFs, in turn, secrete more TGF-β to maintain and increase the activation of fibroblasts (Hawinkels et al., 2014). And the TGF-β can modulate the expression of various EMT markers, such as increased vimentin level and decreased E-cadherin expression, These changes facilitate the transition of tumor cells to a more invasive phenotype, promoting metastasis (Yu et al., 2014). Moreover, at metastatic sites, TGF-β-stimulated CAFs secrete IL-11, which activates the glycoprotein 130-signal transducer and activator of transcription 3 (GP130-STAT3) signaling pathway in tumor cells, this activation enhances the metastasis of CRC cells, ultimately promoting the organ colonization of metastatic CRC (Itatani et al., 2019). Another key factor is Wnt2, a protein secreted by CAFs. Wnt2 activates the classical Wnt pathway in fibroblasts, increases the migration and invasion of CRC cells, and acts in an autocrine manner to promote CRC (Kramer et al., 2017). To summarize, CAFs secrete TGF-β and Wnt2, which in turn trigger a cascade of signaling events that promote CRC cell invasion, migration, and EMT, ultimately leading to metastasis. Targeting these CAF-secreted factors and the metastasis-related signaling pathways could be a promising strategy for inhibiting CRC progression and metastasis.

## 4 Summary and perspectives

CRC is the third most common cause of cancer-related deaths worldwide (Biller and Schrag, 2021). Once metastasized, treatment strategies can be limited, and, if unresectable, outcomes in CRC patients are unfavorable. CAFs, as one of the important components of TME, are considered a crucial therapeutic target due to their involvement in the genesis and progression of tumors (Tauriello, 2023). At present, there have been a number of clinical trials evaluating the therapeutic effects of some drugs targeting biomarkers of CAFs for different cancers, but only two for CRC, and few of these drugs have provided promising results (Zhou and Zheng, 2024). As mentioned above, the origin of CAFs is very complex, resulting many subtypes with different biological functions in tumor progression, including remodeling the ECM, promoting EMT, increasing tumor angiogenesis, suppressing immunity, enhancing and maintaining tumor cell stemness, regulating tumor cell metabolism and metastasis-related signaling pathways. These processes in which CAFs participate in cancer metastasis are not independent and may occur simultaneously. These findings highlight the need for a more comprehensive understanding of the origin and role of CAFs in CRC.

The field faces a significant controversy surrounding the dual nature of CAFs, which typically exhibit tumor-promoting properties but can also exhibit potential tumor-suppressive functions. This dualism poses a substantial challenge for targeted therapy, as it necessitates the ability to selectively target the tumor-promoting CAFs without inadvertently disrupting their potential tumor-suppressive CAFs. Enhancing our understanding of CAF subtypes holds the key to overcoming this challenge more effectively.

While advances have been made in the study of CAFs, there are still several knowledge gaps that need to be addressed. The precise molecular pathways underlying the differentiation of CAFs from their diverse precursor cells have yet to be fully elucidated. Additionally, given high heterogeneity of CAFs, there is currently no single biomarker that is completely specific for identifying and isolating CAFs from tumor tissues, and identifying reliable biomarkers to distinguish different CAFs subtypes remains a critical challenge. Moreover, the correlation between the origins of CAFs in CRC and their impact on CAFs function is still uncertain. Addressing these gaps, which future research should focus on, is essential for enhancing our understanding of CAF roles and develop effective therapeutic strategies. Theoretically, CAF heterogeneity and their multifunctional roles should be deeperly understood. Technically, the development of more advanced *in vivo* models that closely mimic the human TME is crucial for elucidating the origin and function of CAFs. Such models will be invaluable for studying the dynamic interactions between CAFs and other components of the tumor stroma. Moreover, the development of highly specific biomarkers for different CAF subtypes is essential for the improvement targeted therapies.

In conclusion, CAFs, characterized by their high heterogeneity and diverse functions, play vital roles in the invasion and metastasis of CRC. As novel techniques like scRNA-seq continue to advance, the comprehensive characterisation of CAFs will become increasingly clear. This deeper understangding will pave the way for the development of new strategies specifically targeting CAFs, offering promising avenues for CRC treatment in the future.
